# A comprehensive integrated dataset on Brazilian health facilities: from 2005 to 2021

**DOI:** 10.1186/s13104-023-06422-y

**Published:** 2023-07-20

**Authors:** Rebecca Salles, Victor de Paula Dornellas Ribeiro, Raquel Gritz, Carlos Leonardo Souza Cardoso, Balthazar Paixão, Lucas Carraro, Sérgio Ricardo de Borba Cruz, Vinicius Kreischer, Carmen Lúcia Corrêa Bonifácio, Raphael de Freitas Saldanha, Gabriel Souto, Matheus Miloski, Leandro Zirondi, Gizelton Pereira Alencar, Ariane Camilo Pinheiro Alves, Nelson Niero Neto, Jefferson da Costa Lima, Marcel Pedroso, Ronaldo Fernandes Santos Alves, Patricia de Moraes Mello Boccolini, Cristiano Siqueira Boccolini

**Affiliations:** 1 Platform of Data Science applied to Health (PCDaS), Institute of Scientific and Technological Communication and Information on Health (ICICT), Oswaldo Cruz Foundation (Fiocruz), Rio de Janeiro, Brazil; 2 Federal Center for Technological Education of Rio de Janeiro, CEFET/RJ, Rio de Janeiro, Brazil; 3 Data Extreme Lab, National Laboratory for Scientific Computing, DEXL Lab/LNCC, Petrópolis, Brazil; 4 Institute of Scientific and Technological Communication and Information on Health (ICICT), Oswaldo Cruz Foundation (Fiocruz), Rio de Janeiro, Brazil; 5 Center for Information, Public Policies and Social Inclusion, NIPPIS, FMP/UNIFASE, Petrópolis, Brazil; 6 Federal University of Rio de Janeiro, UFRJ, Rio de Janeiro, Brazil; 7 School of Public Health, University of São Paulo, FSP/USP, São Paulo, Brazil; 8 National Laboratory for Scientific Computing, LNCC, Petrópolis, Brazil

**Keywords:** Health facilities, Data integration, Healthcare system

## Abstract

**Objectives:**

The National Registry of Healthcare Facilities is a system with the registry of every healthcare facility in Brazil with information on the capacity building and healthcare workforce regarding its public or private nature. Despite being publicly available, it can only be accessed in separated disjoint tables, with different primary units of analysis. The objective is to offer an interoperable dataset containing monthly data from 2005 to 2021 with information on healthcare facilities, including their physical and human resources, services and teams, enriched with municipal information.

**Data description:**

Database with historical data and geographic information for each health facility in Brazil. It is composed by 5 distinct tables, organized according to combinations of time, space, and types of resources, services and teams. This database opens up a range of possibilities for research topics, from case studies in a single health facility and period, analysis of a group of health facilities with characteristics of interest, to a broader study using the entire dataset and aggregated data by municipality. Furthermore, the fact that there is a row for each health facility/month/year facilitates the integration with other datasets from the Brazilian healthcare system. In addition to being a potential object of study in the health area, the dataset is also convenient in data science, especially for studies focused on time series.

## Objective

Approximately half the world’s population lacks access to healthcare services, medicines, and vaccination [1]. Brazil includes universal health coverage in its Constitution, which is operated through the Unified National Health System (“Sistema Único de Saúde” in Portuguese) [2], aligned with the target 3.8 of the Sustainable Development Goals [3]. The National Registry of Healthcare Facilities (“Cadastro Nacional de Estabelecimentos de Saúde”; or CNES, in Portuguese) is a system with the registry of every healthcare facility in Brazil with information on the capacity building and healthcare workforce regarding its public or private nature [4]. The CNES must be fed monthly by each healthcare unit, providing updated knowledge about the existing healthcare network to assist in health planning and effective and efficient management. Public consultations can be made on the CNES system (http://cnes.datasus.gov.br/), but the outputs will be provided in separated tables for each system dimension: healthcare facility’ general information, physical resources, human resources, services, and health teams. Every dataset dimensions has between 49,989,883 and 55,355,133 registers. Moreover, the number of attributes varies between 451 and 849.

Our objective is to provide a machine-readable and interoperable dataset on the CNES dimensions. This dataset will support studies that describe the Brazilian healthcare situation, that employ healthcare dimensions as confounders in ecological designs, or studies on the impact of public policies on health outcomes. Data from CNES are currently only available through tabular sets or via FTP and in a disaggregated way. The presented datasets make them available to the community, in a standardized way and following the FAIR principles. They provide unified access to the datasets and enrichments with geospatial information, which allows analysis in total granularity (accessing Brazilian health facilities), and spatial and temporal aggregated data.

## Data description

The datasets created contain the different dimensions of the National Registry of Health Facilities (CNES), which are: physical resources (beds and equipments), human resources (health professionals), services and health teams. The dataset and its related files are available for download, as shown in Table 1. Each of these datasets results from a construction method with the following steps: (i) data extraction, (ii) data integration, (iii) data mapping, (iv) reshaping data, and (v) data enrichment. The criteria adopted for data management were based on detailed studies of the dataset, and the support of specialists in the field.^1^

i. The datasets were extracted from DATASUS, and each one represents a different dimension of health facilities, with records of physical resources, such as types of existing beds and amounts of equipments existing and in use in the health facilities, as well as the health services offered, numbers of professionals and types of health teams. For this, data on health facilities (ST), complementary data from the CNES (DC), and data from each dimension were extracted. In addition, data extraction was performed over time (months and years) and in geographic space (federative units), with the observation of health units.

ii. The datasets developed are the result of integrating the three types of databases previously extracted: complementary data, health facilities, and dimension, aggregated by competence date (months and years) and federative unit in Brazil.

iii. The data mapping step aims to create variables where, for each numeric value of a source variable, a categorical definition value is created.

iv. To reshape data, the values in indexes/columns are organized, creating new datasets. From there, the treatment of invalid values is performed.

v. Finally, the dataset was enriched with information about the municipalities, states, and regions obtained from the DATASUS FTP server.^2^ The attributes resulting from this process are the coordinates, area, regional health codes, and other Boolean attributes, indicating if the municipality is the state’s capital or border, for example. The data dictionary^3^ presents a full description. All of these steps are explained in details and can be reproduced for the CNES Teams dataset, as an example, with the Jupyter Notebook file available at https://doi.org/10.7303/syn34388051.1.

**Table 1:** Overview of data files/datasets

Label	Name of data file/dataset	File type (file extension)	Data repository and identifier	References
Dataset 1	CNES Beds (50M records, 451 attributes)	Compressed files (.zip)	Synapse: https://doi.org/10.7303/syn32209177.1	[5]
Dataset 2	CNES Equipments (50M records, 562 attributes)	Compressed files (.zip)	Synapse: https://doi.org/10.7303/syn32208612.1	[6]
Dataset 3	CNES Professionals (49M records, 656 attributes)	Compressed files (.zip)	Synapse: https://doi.org/10.7303/syn32210090.1	[7]
Dataset 4	CNES Services (49M records, 849 attributes)	Compressed files (.zip)	Synapse: https://doi.org/10.7303/syn32211006.1	[8]
Dataset 5	CNES Teams (55M records, 694 attributes)	Compressed files (.zip)	Synapse: https://doi.org/10.7303/syn32207908.1	[9]
Data file 1	cnes_beds_dictionary	Delimited text (.csv)	Synapse: https://doi.org/10.7303/syn32167629.1	[10]
Data file 2	cnes_equipments_dictionary	Delimited text (.csv)	Synapse: https://doi.org/10.7303/syn32167630.1	[11]
Data file 3	cnes_professionals_dictionary	Delimited text (.csv)	Synapse: https://doi.org/10.7303/syn32167631.1	[12]
Data file 4	cnes_services_dictionary	Delimited text (.csv)	Synapse: https://doi.org/10.7303/syn32167632.1	[13]
Data file 5	cnes_team_dictionary	Delimited text (.csv)	Synapse: https://doi.org/10.7303/syn32167633.1	[14]
Data file 6	CNES_ETL_report	Portable document (.pdf)	Synapse: https://doi.org/10.7303/syn34388050.2	[15]
Data file 7	ETL_example_CNES_EP	Jupyter notebook (.ipynb)	Synapse: https://doi.org/10.7303/syn34388051.1	[16]

The constructed datasets provide information about the CNES in a machine-readable, ready-to-use format. They allow evaluating and understanding the spatial distribution and temporal evolution of Brazil’s private and/or public health infrastructure. Furthermore, they contribute to the aggregation of ecological studies, with the municipality as the primary unit of analysis, helping in studies that seek to observe particular public health aspects such as hospitalization for diarrhea or pneumonia, vaccine coverage, and preventable infant mortality, for example [17].

## Limitations

- As the feeding of this data is the responsibility of the health facilities themselves, errors and inconsistencies in the registration of data may occur, as well as the discontinuity of the same. Thus, despite the data being monthly, its quality is not guaranteed on a monthly basis.
- When using these data, consider that the first years were periods of transition/adaptation of the system, as well as the correct use and registration of health facilities in the CNES, so inconsistencies and duplicities may occur, mainly in the first 3 years.
- There are variables which go into disuse, therefore their feeding are discontinued.
- The year 2005 is included in the dataset from the eighth month onwards, and as it represents the beginning of the CNES system, its data are not fully complete in the first months.

## Data Availability

The CNES datasets corresponding to beds [5], equipments [6], professionals [7], services [8], and teams [9]; the CNES data dictionaries related to beds [10], equipments [11], professionals [12], services [13], and teams [14], as well as the CNES ETL process report [15] and an descriptive example of the CNES teams ETL process [16] is freely and openly available on the Synapse repository at https://doi.org/10.7303/syn26343275. Anyone can browse the content on the Synapse website, but you must register for an account using your email address to download the files and datasets. Please see Table 1 for details and links to the data.

## Footnotes

1 Description of the construction of the datasets is available at https://doi.org/10.7303/syn26343275.

2 Available at ftp://ftp.datasus.gov.br/dissemin/publicos/SINASC/NOV/TABELAS/.

3 Available at https://doi.org/10.7303/syn26343275.
